# Supplementary material for: Control of primary metabolism by a virulence regulatory network promotes robustness in a plant pathogen
Source: Nat Commun. 2018 Jan 29;9:418. doi: 10.1038/s41467-017-02660-4 (PMC5788922; doi:10.1038/s41467-017-02660-4)
Supplement: Supplementary file 1 — Supplementary Information [file 41467_2017_2660_MOESM1_ESM.pdf]

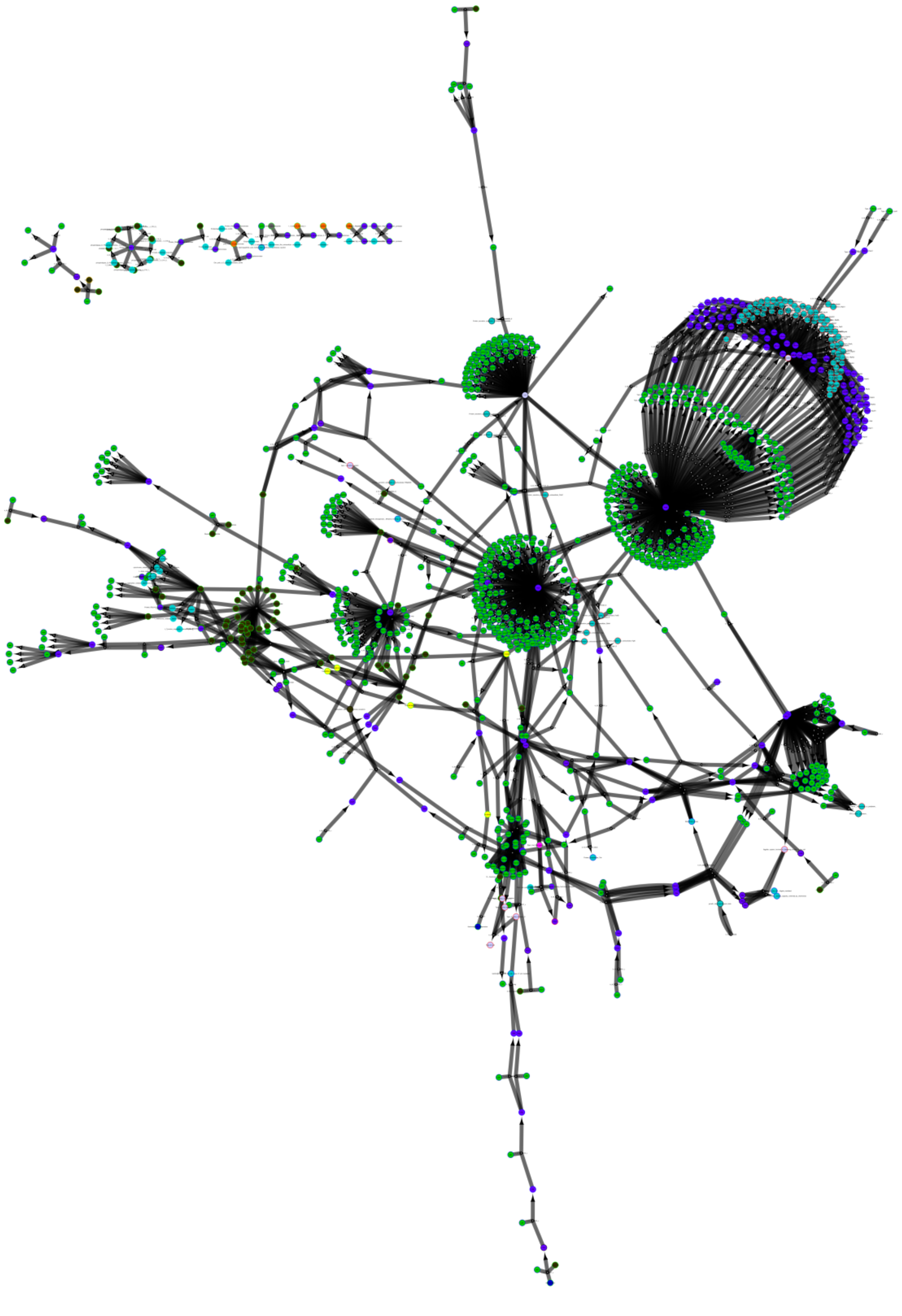

Supplementary Figure 1. Map of the Virulence Regulatory Network (VRN), iRP1443REG. The map was drawn using the software Cytoscape. Details about nodes (VRN components) and edges (interactions between those components) can be found in the Supplementary Data 1.

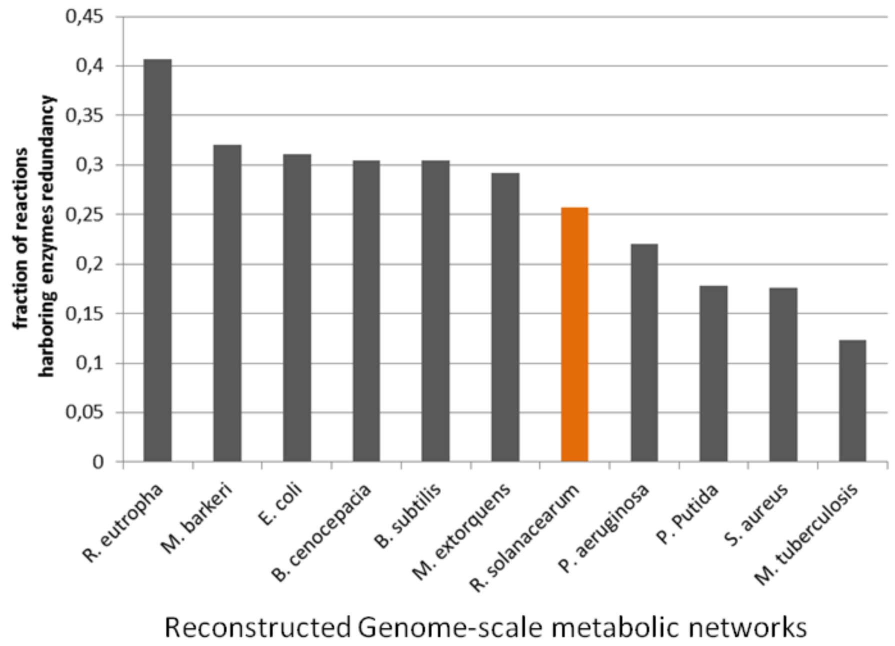

Supplementary Figure 2. Proportion of reactions harboring enzymes redundancy in reconstructed genome-scale networks. Enzymes classified as redundant correspond to enzyme with a GPR in the metabolic network harboring at least one of the logical function “or”. This means that at least one subunit of the enzyme can be substituted by a protein coming from another gene.

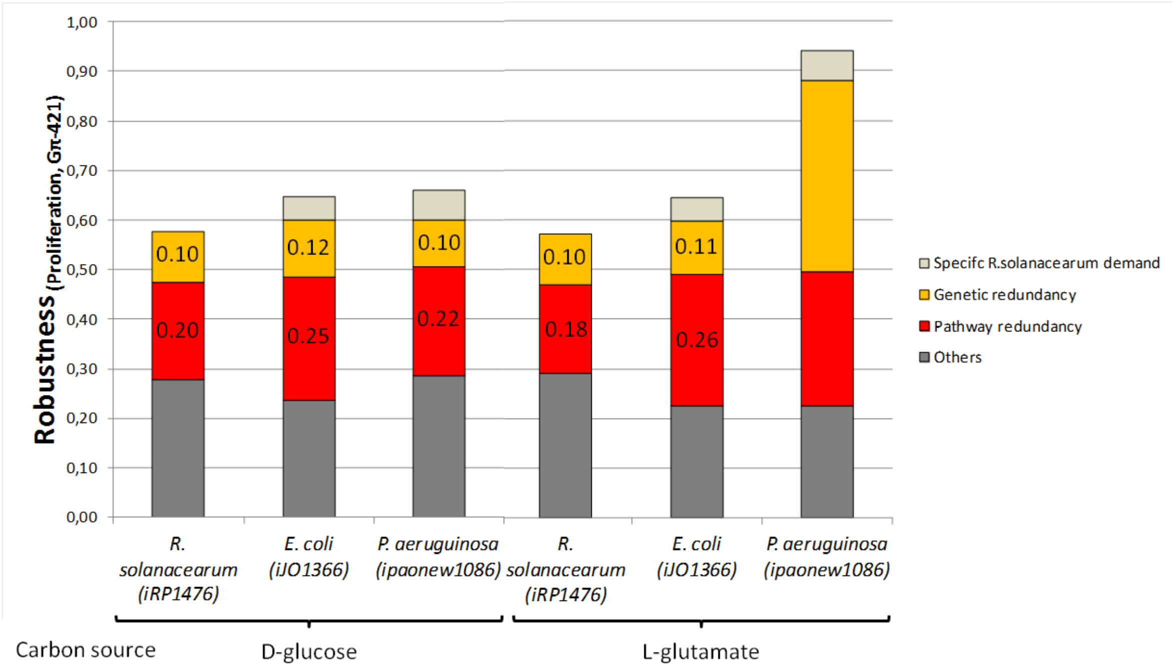

Supplementary Figure 3. Robustness of the *R. solanacearum*, *E. coli* and *P. aeruginosa* metabolic networks with respect to the same set of genetic perturbations. Robustness was calculated from the same genetic perturbation using a set of 421 orthologs. Specific *R. solanacearum* demand corresponds to genes involved in the biomass function in *R. solanacearum* but not in the biomass function of the two others.

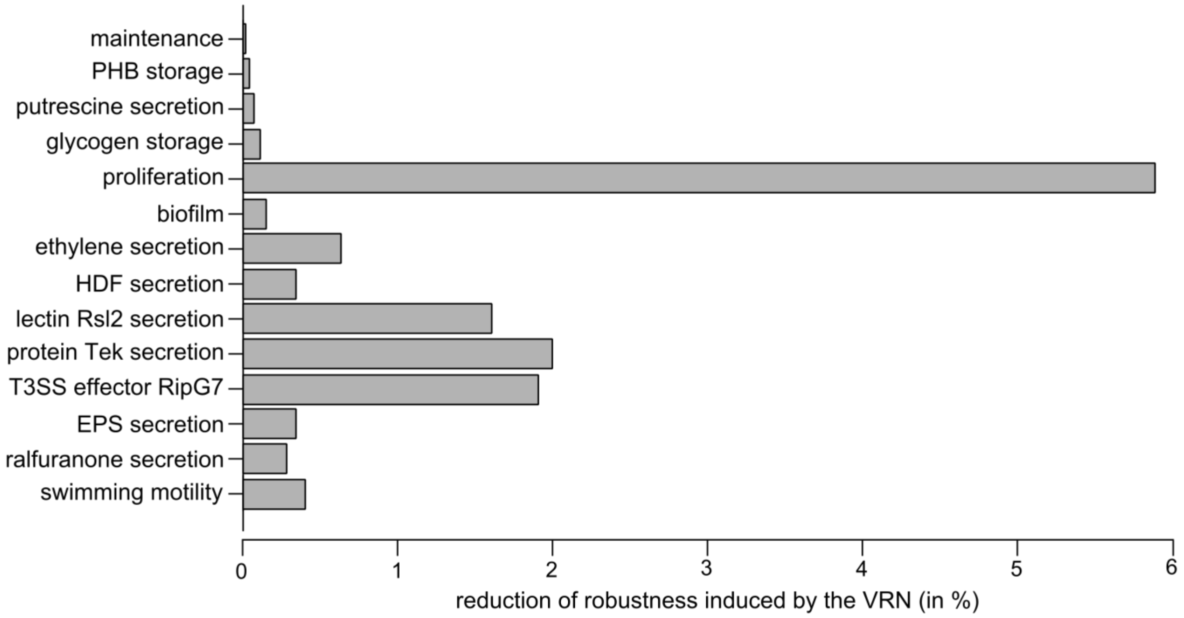

Supplementary Figure 4. Impact of the VRN control on metabolism on the phenotypic robustness with respect to internal perturbation. The BECO analysis was conducted on the 14 selected phenotypes, under the 16 environmental conditions and using internal perturbations corresponding to genes and gene products loss of function. The reduction of robustness is given in robustness metric (R) and corresponds to the percentage of robustness reduction.

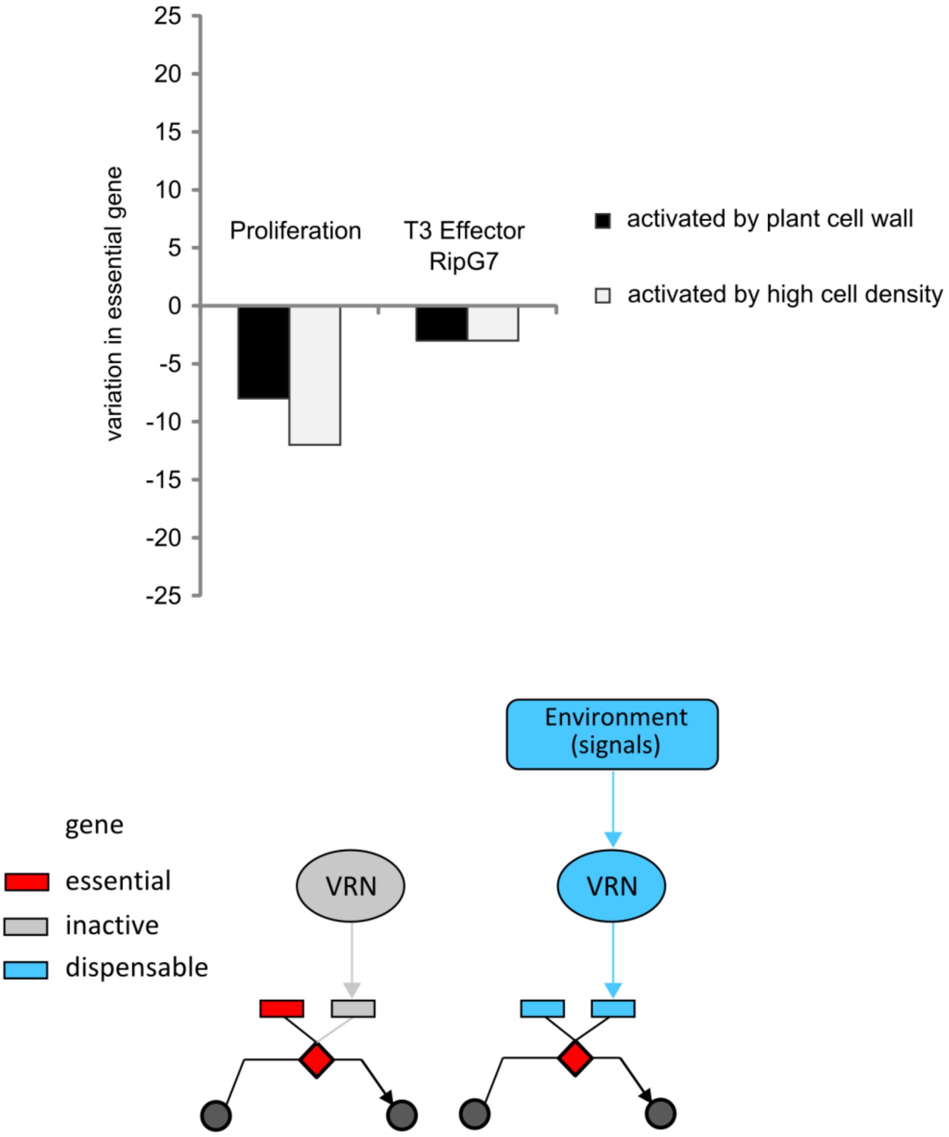

Supplementary Figure 5. Variation in essential genes upon activation of the VRN. Activation of the VRN by signals encounter *in planta* by the pathogen, like plant cell wall components or high cell density, decreases the number of essential genes.

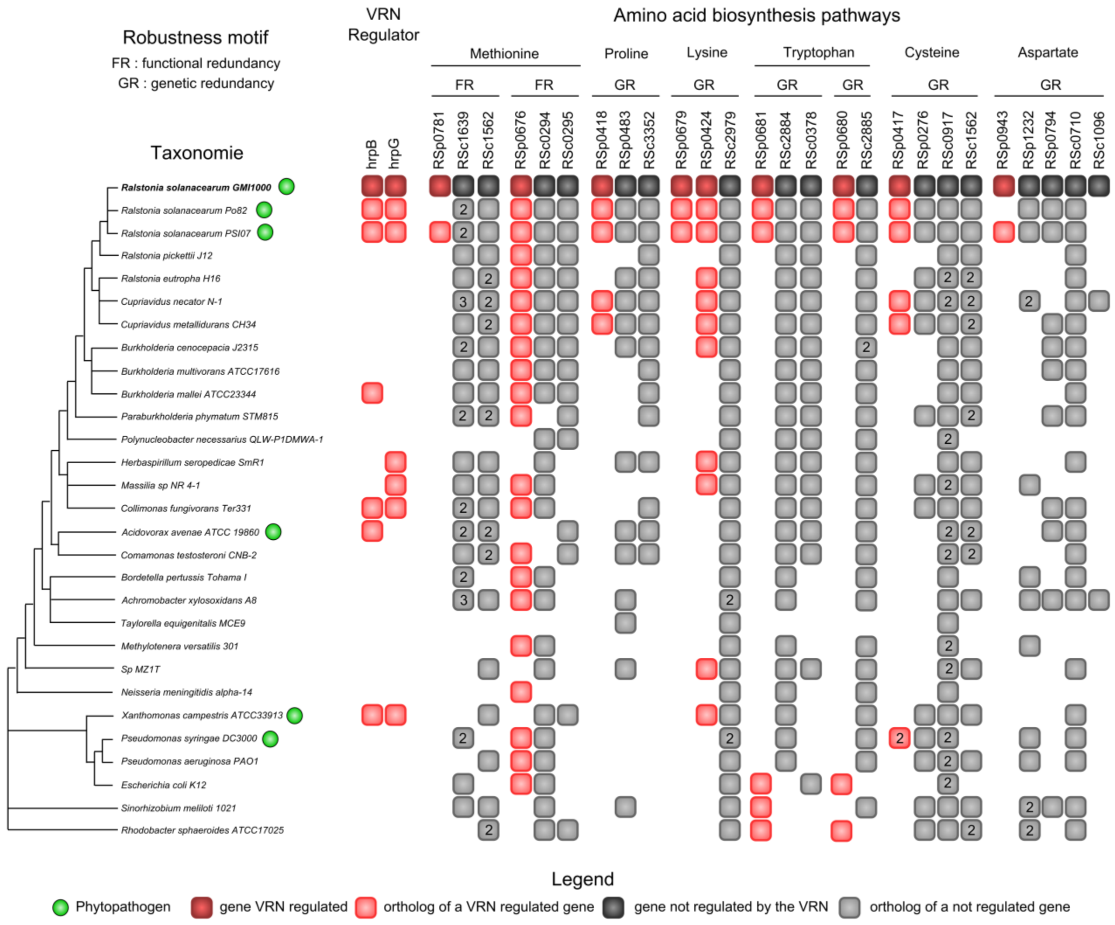

Supplementary Figure 6. Orthology analysis of VRN-regulated and VRN-unregulated genes which provide robustness within the amino acids biosynthesis pathways. Presence of orthologs of the *R. solanacearum* genes in the various taxa is displayed by boxes. Number in the boxes corresponds to the number of paralogs present in the organism. Closest orthologs of a VRN-regulated gene are colored in red whereas the closest orthologs of a VRN-independent one are in grey. In the case of two genes belonging to the same orthology group, the closest ortholog to a *R. solanacearum* gene was defined as the gene showing the highest blast score to a *R. solanacearum* gene.

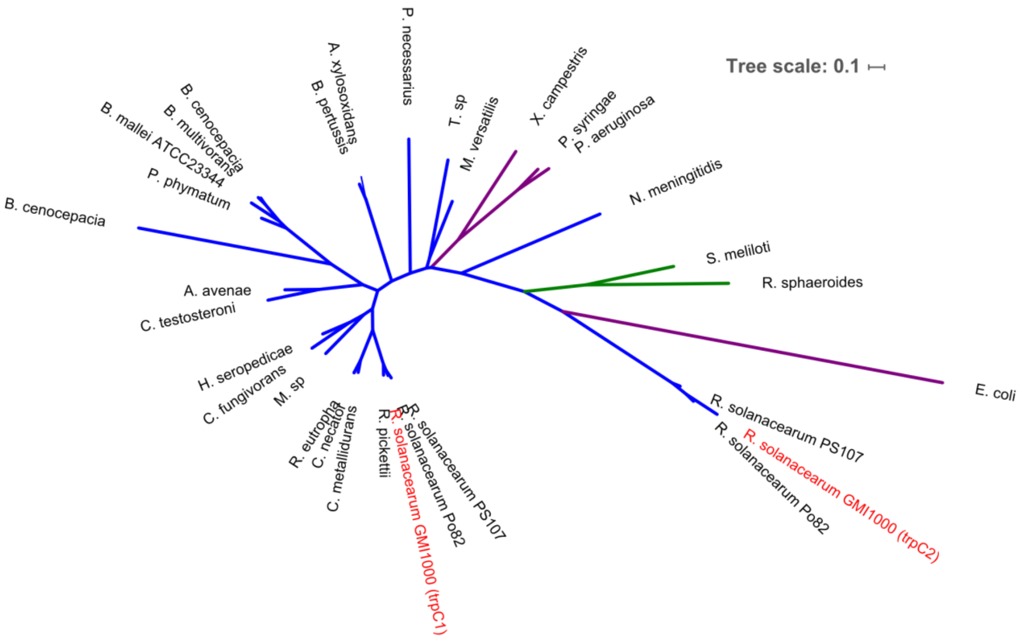

Supplementary Figure 7. Phylogeny analysis of the *trpC1* and *trpC2* orthologs group. Tree scale corresponds to 0.1 mutation.

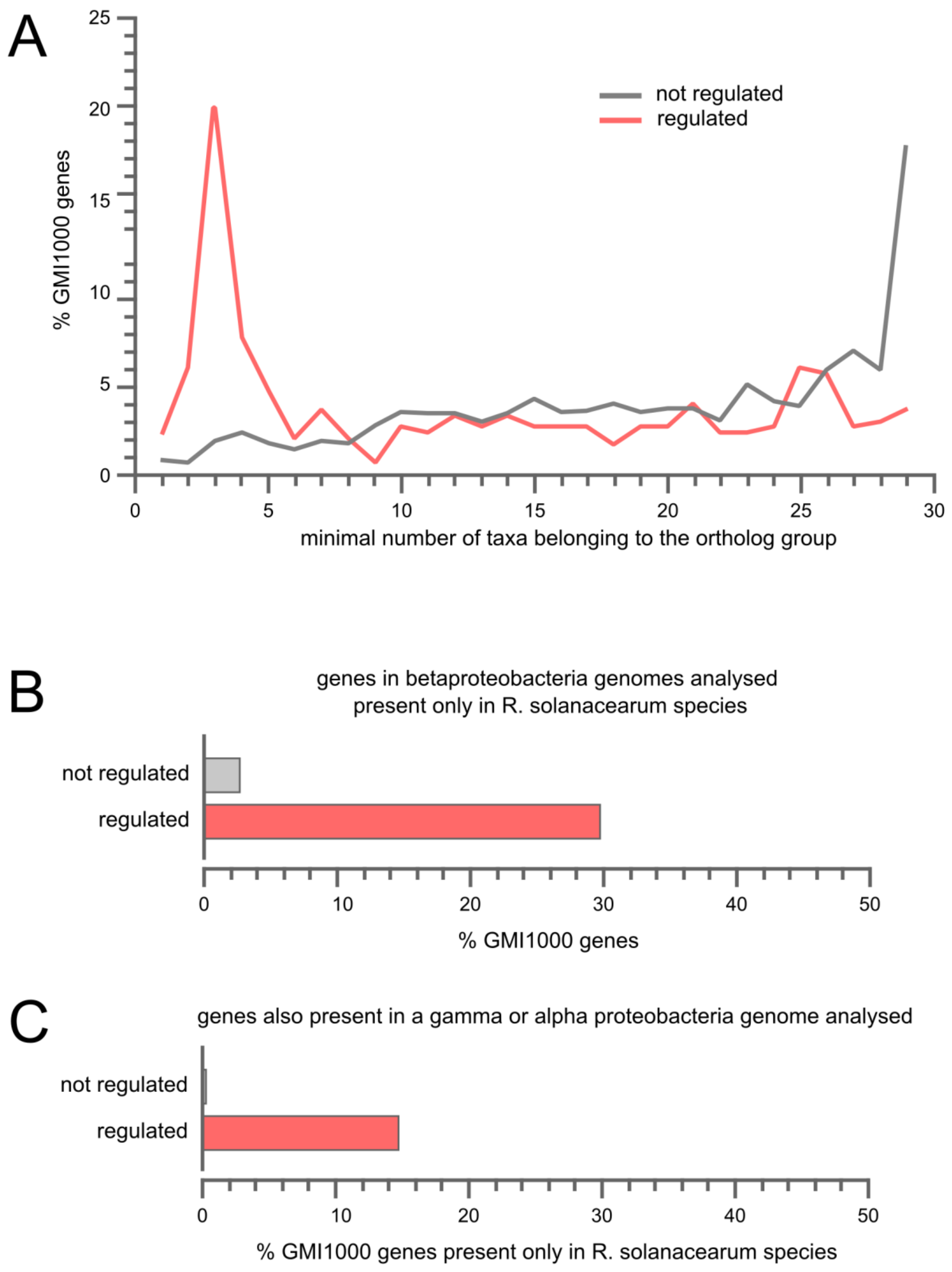

Supplementary Figure 8. Taxonomy distribution of the VRN-regulated metabolic genes. (**A**) % of metabolic genes by their number of taxa to which their orthologs belongs. 28 species were analyzed, see figure 6 for the list. In red, VRN-regulated genes (305 genes); in grey, genes not regulated by the VRN (1122 genes). (**B**) % of genes unique to the *R. solanacearum* species in the beta-proteobacteria class. (**C**) % of genes unique to the *R. solanacearum* species among beta-proteobacteria but present in the alpha and gamma taxons investigated.

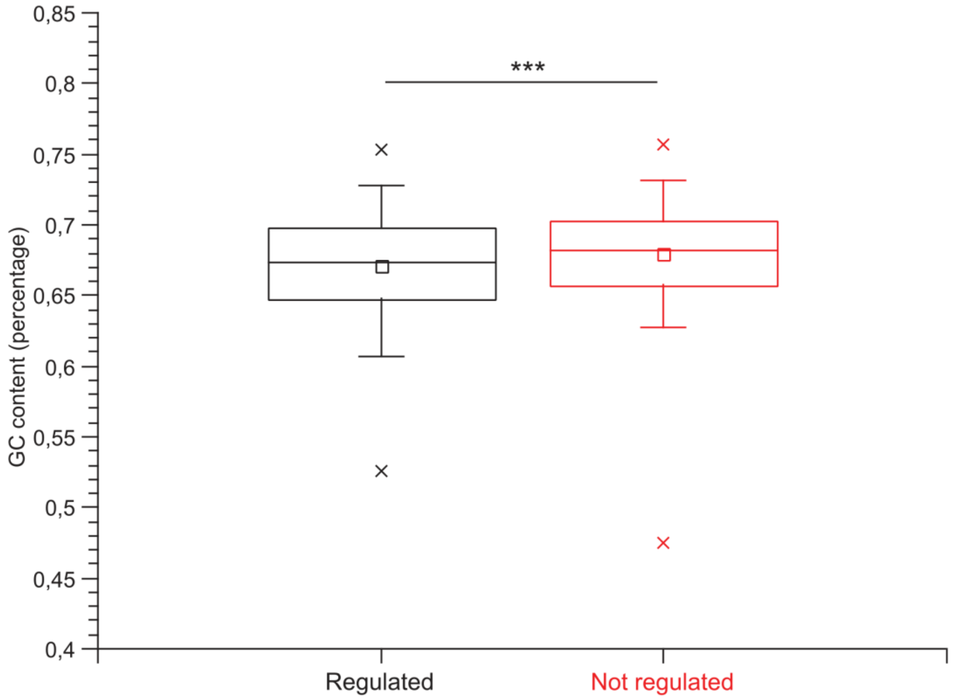

Supplementary Figure 9. GC% content in VRN-regulated genes and VRN-independent genes. Analysis made on 305 VRN-regulated genes (in black) and 1122 VRN-independent genes (in red). ***, significance of the statistical test p-value $3{\cdot}10^{-4}$.

SUPPLEMENTARY NOTE 1

Integration of the metabolic and the regulatory network

In order to better understand the influence of the regulatory network on the metabolic network behaviour, it was indispensable to model the interactions between the networks. Like in other methods [1-3], the links between regulatory and metabolic networks are established by constraining the fluxes of the latter by solving the former. Reactions in the metabolic network are linked to the regulatory network by the gene-protein-reaction associations (GPR), by the metabolites they involve or can be themselves outputs of the regulatory network.

Computing the optimal value of an objective function given a metabolic network (M) and a regulatory network (R) for which the initial values have been set is performed in four steps:

1) Integration of environmental constraints in the regulatory network which correspond to the initial node states. The translation of continuous intervals to discrete values is made from notes added by the modeller in the SBML-qual [4] input file.
2) Identification of the regulatory network steady state by finding an attractor (see above)
3) Translation of the regulatory network steady state into constraints for the FBA
4) Phenotype prediction by optimising an objective function

We integrated this framework in FlexFlux [5] a java library that easily allows integrating flux balance analysis and regulatory network analysis. We used FlexFlux for all computational analyses of this study. More detail can be found on the online documentation of FlexFlux [5].

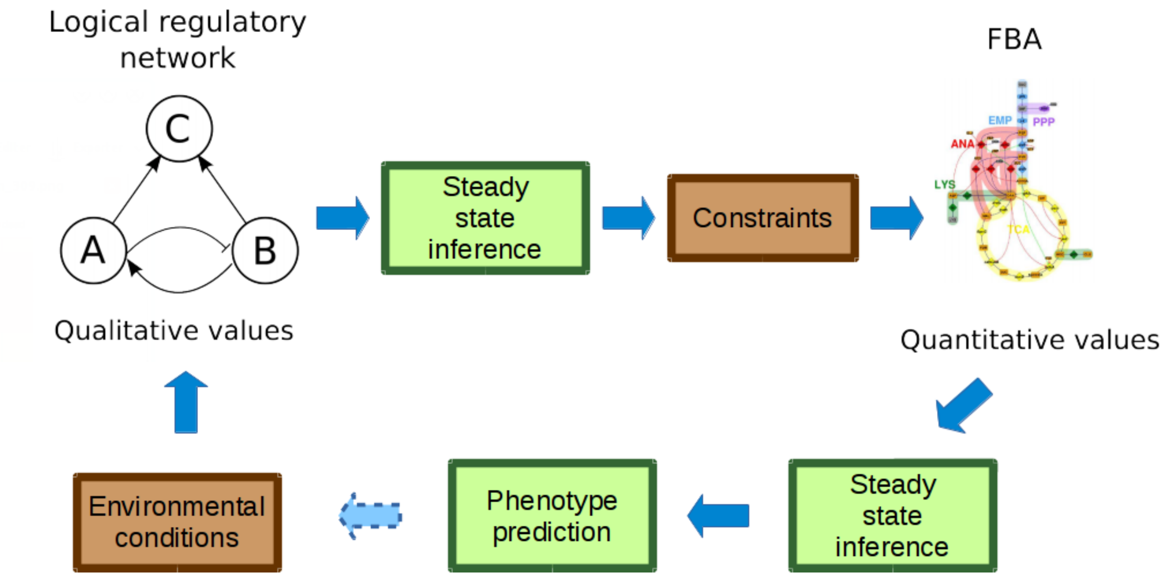

Supplementary Figure 10. Links between regulatory and metabolic network analyses in FlexFlux. The dashed arrow corresponds to the change of environmental conditions by the output of the FBA that can be used in the dynamic regulated FBA function of FlexFlux [5].

SUPPLEMENTARY NOTE 2

Prediction of *in planta* transcriptome

Supplementary Figure 11 illustrates the validation process of the gene differential expression prediction detailed in the Material & Methods of the main paper. The pipe-line allows comparison between experimental data with simulated data. To do so, both data sets are converted into 3 classes: differentially up regulated, differentially down regulated, and not differentially expressed.

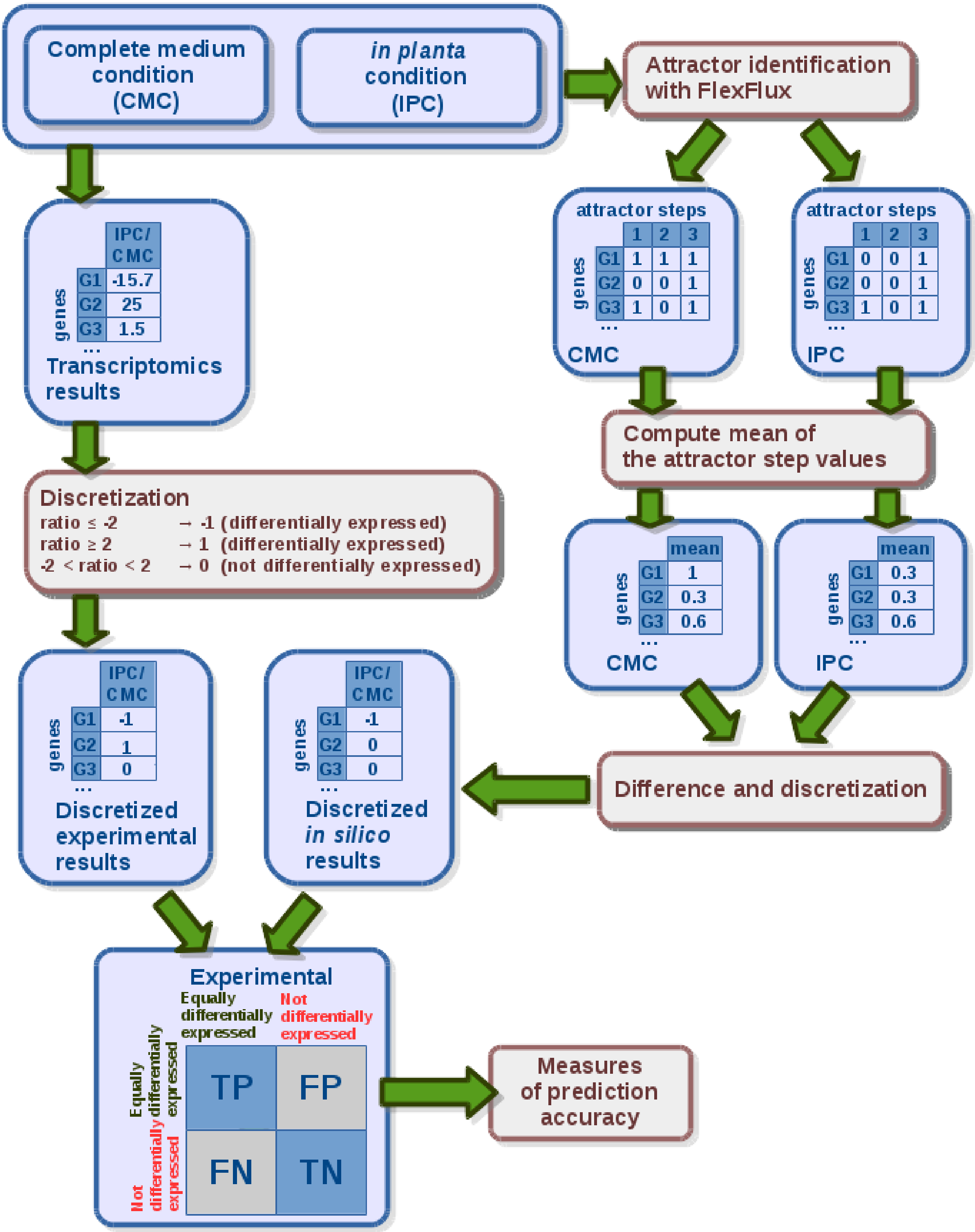

Supplementary Figure 11. Gene differential expression prediction.

SUPPLEMENTARY NOTE 3

Validation of the gene expression predictability by the VRN

Analysis of the discrepancy between the network model prediction and experimental data concerned almost exclusively the *hrpB* regulatory gene targets, since only half of them were found to be experimentally induced differentially *in planta* [6]. However, this discrepancy was already observed in a previous experimental study performed under a different bacterial growth condition [7], see Supplementary Figure 12.

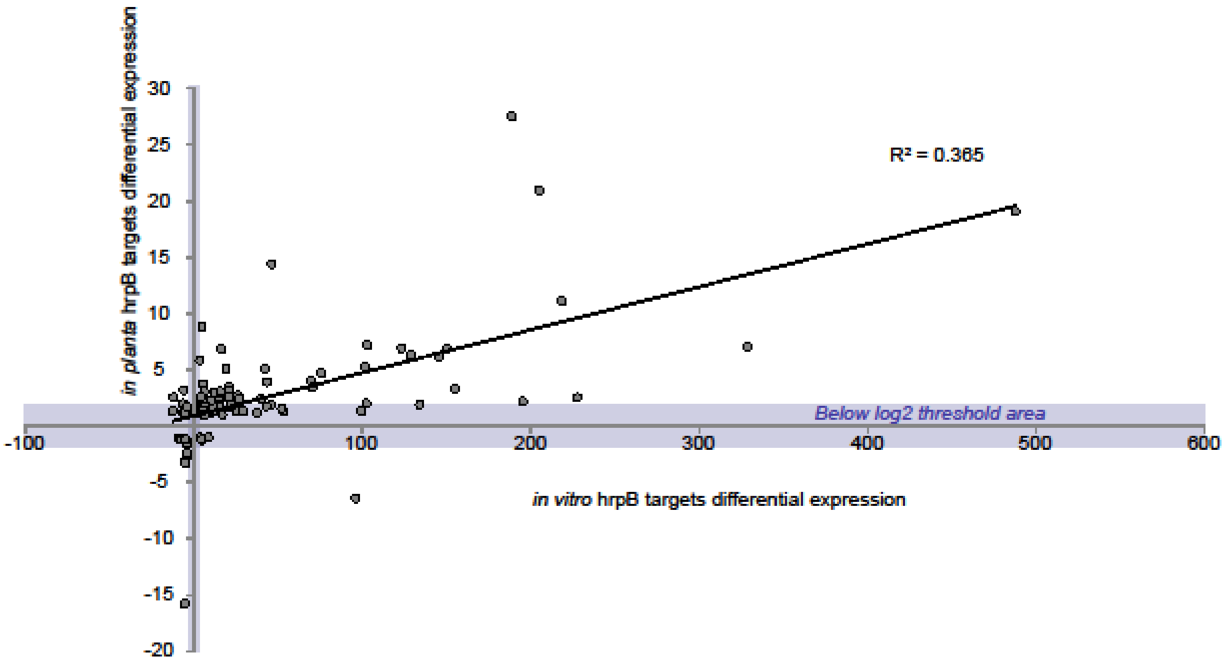

Supplementary Figure 12. Comparison of HrpB targets expression profiles between the *in planta* transcriptome analysis and *in vitro* analysis. The *in planta* transcriptome dataset was extracted from Jacobs et al. (2012)[6] and the *in vitro* (synthetic inducible medium) dataset was extracted from Occhialini et al. (2005)[7]. R^2 corresponds to the linear regression display as black line.

SUPPLEMENTARY NOTE 4

Comparison between *in silico* knock-out phenotype predictions and experimental Tn5 insertion mutant phenotypes

Experimental TN5 insertion mutants were converted to a Boolean matrix with two columns corresponding to glucose and glutamate. Each row corresponds to a mutant and the Boolean value in a cell is 1 if growth has been identified for a mutant in the corresponding condition (Supplementary Figure 13, left part), 0 otherwise. Thanks to the genome annotation (https://iant.toulouse.inra.fr/bacteria/annotation/cgi/ralso.cgi), links between metabolic genes and mutants have been established and converted into a Boolean matrix indicating for each mutant which genes have been knocked out. Then, we used the FlexFlux [5] BECO function to translate this Boolean matrix in sets of flux constraints and to predict the presence or absence of growth of each mutant in glucose condition and in glutamate condition. The results were finally converted into a Boolean matrix (Supplementary Figure 13, right part) that has been compared to the matrix computed from experiments. The comparison has been used to make corrections into the model and to measure the accuracy of the model predictions. The number of true positive predictions (TP) corresponds here to the number of conditions for which both experimental and *in silico* methods found an essential gene (growth=0). The number of positive predictions (P) corresponds to the number of conditions for which the *in silico* method found an essential gene. The number of false negative predictions (FN) corresponds to the number of conditions for which the *in silico* method found a positive growth while the experimental method found a null growth.

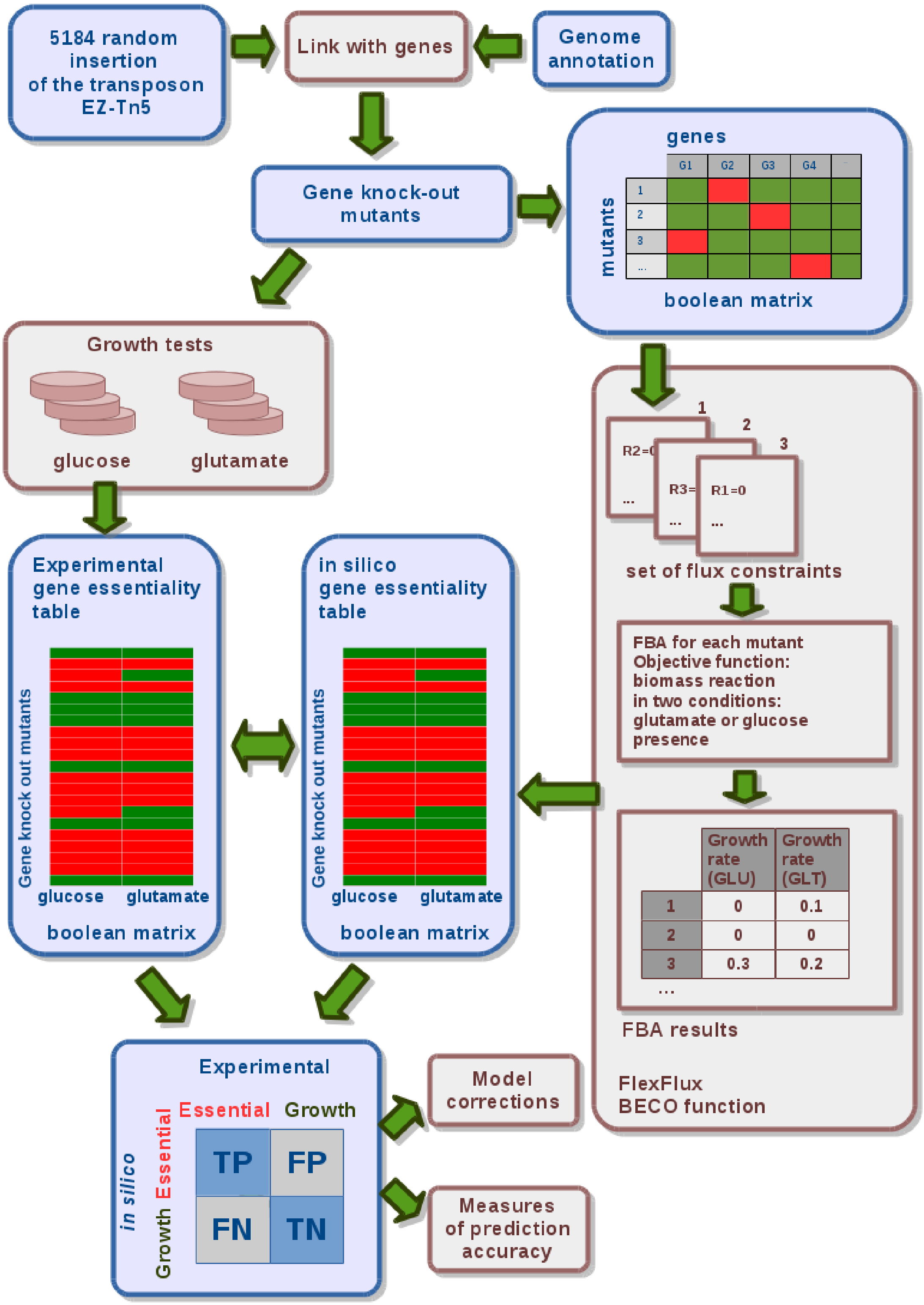

Supplementary Figure 13. Metabolic network validations by comparing experimental and *in silico* knock out phenotypes

SUPPLEMENTARY NOTE 5

Classification of reactions and genes

In order to identify sources of phenotypic robustness (Figure 1 of the article), it was important to classify the reactions and the genes considering their effect on the realisation of the objective value. For this, we modified an algorithm already described by [8]. Instead of considering only genes, we first start by the classification of the reactions themselves and use this for classifying the genes. Moreover, we added one category to the classification described in [8] that were not taken into account: objective independent reactions correspond to reactions that have no effect on the objective value.

The whole pipeline for classifying reactions is presented in Supplementary Figure 14. At last, the algorithm to classify genes from the classification of the reactions and the gene reaction links is described in Supplementary Figure 15. These algorithms are implemented in the function "Classification" of FlexFlux and used in the BECO function described in the Material and Methods section and detailed in the following.

The reaction categories are:

- Essential reactions whose the deletion makes the objective function's value to be null
- Zero flux reactions can't carry flux, i.e. their flux value is always 0
- MLE (Metabolically less Efficient Reactions) enables objective function but not in an optimal way
- ELE (Enzymatically less Efficient Reactions) makes the objective function's value to be optimal but by using more enzymes than other optimal solutions
- Objective Independent Reactions can carry flux but have no effect on the objective function
- pFBA optima reactions (OPT) makes the objective function's value to be optimal but by using a minimal number of enzymes

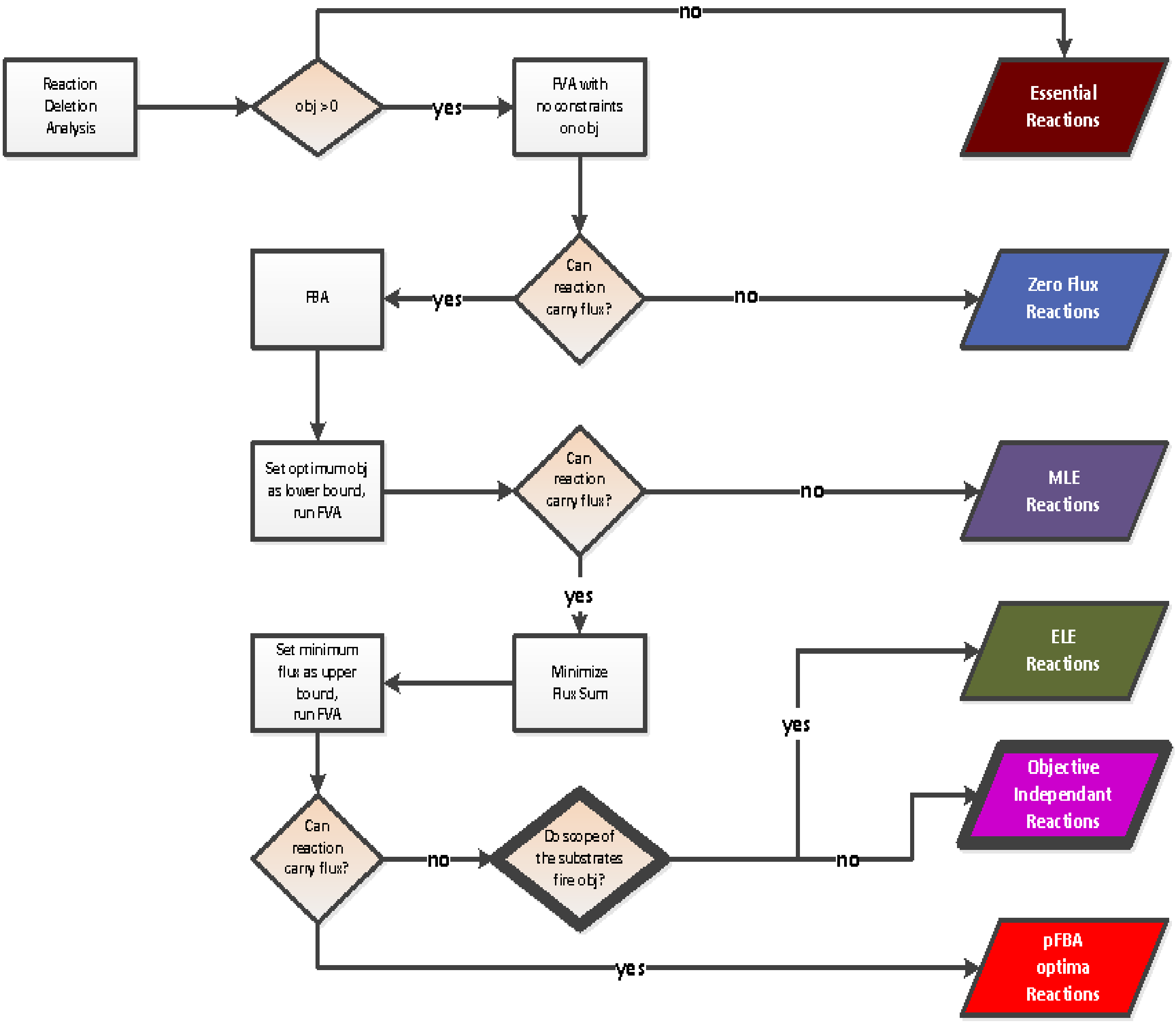

Supplementary Figure 14. Classification of the reactions. In bold the steps absent from the classification described in [8].

Biological Entity Clustering by their contribution to the Objective function (BECO)

The method called Biological Entity Clustering by their contribution to the Objective function (BECO) proceeds as following.

For each objective function (OB), for each condition: i) a reaction deletion analysis is performed and reactions KO leading to OB=0 are classified as essential; ii) then, a FVA is performed without constraints on the objective function in order to identify the reactions never carrying fluxes. They are classified as Zero Flux Reactions (ZFR); iii) then, a FBA is performed to obtain optimum value for the objective function and a FVA is run at 100% of the OB as constraint. The reactions not carrying

fluxes are classified as metabolically less efficient (MLE) reaction; iv) then, a FBA with 100% of the OB as constraint is run with minimization of the sum of fluxes. Then, a new FVA is run with 100% of OB and 100% of the minimal flux sum as constraints. The reactions which carry fluxes are classified as optimal reactions. The forward propagation algorithm [9] is used to compute the scope (sub-network highlighted by the forward propagation algorithm) of the substrates of the reactions not carrying fluxes. If the scope contains the reactions corresponding to the objective function, the reactions are classified as enzymatically less efficient (ELE) reactions. If not, they are classified as objective independent reactions (OIR). iv) Then, the genes are classified depending on the classification of the reaction for which they are involved in the catalysis via the GPR (Supplementary Figure 15).

ClassifyGenesFromReactionClasses algorithm

Inputs:
- Network ***N*** with gene protein reaction links (GPR)
- Reaction classes:
    - ***R_ES*** : essential reactions
    - ***R_ZF*** : zero flux reactions
    - ***R_MLE*** : metabolically less efficient reactions
    - ***R_ELE*** : enzymatically less efficient reactions
    - ***R_OIR*** : objective independent reactions
    - ***R_OPT*** : pFBA optima reactions
- ***O*** : objective function

Output:
- Gene classes:
    - ***G_ES*** : essential genes
    - ***G_ZF*** : zero flux genes
    - ***G_MLE*** : metabolically less efficient genes
    - ***G_ELE*** : enzymatically less efficient genes
    - ***G_OIR*** : objective independent genes
    - ***G_OPT*** : pFBA optima genes

Algorithm:

```
FOREACH G in genes(N)

        reactions = reactions catalysed by G

        // First chech if the gene is essential by FBA
        optValue  = GeneDeletionAnalysis(O, G)
        if(optValue == 0)
                PUT G in G_ES
        ELSE IF(reactions ∩ R_OPT ≠ {})
                PUT G in G_OPT
        ELSE IF(reactions ∩ R_MLE ≠ {})
                PUT G in G_MLE
        ELSE IF(reactions ∩ R_ELE ≠ {})
                PUT G in G_ELE
        ELSE IF(reactions ∩ R_OIR≠ {})
                PUT G in G_OIR
        ELSE IF(reactions ∩ R_ZF≠ {})
                PUT G in G_ZF
```

Supplementary Figure 15. Classification of the genes from the classification of the reactions

SUPPLEMENTARY NOTE 6

Level of genetic redundancy and functional redundancy within primary metabolism is similar in *R. solanacearum* compare to other bacteria.

In a first analysis, we sought to evaluate the extent of the functional redundancy (pathway redundancy plus genetic redundancy) providing robustness within the metabolic network of *R. solanacearu*m. Indeed, this prior analysis is required to further deconvoluate if there is some specificity of the metabolic network of *Ralstonia solanacearum* in term of robustness before analysis of the virulence regulatory network contribution. Hence, we quantified the genetic redundancy of enzymes, i.e. the proportion of the reactions that can be catalysed by alternative enzymes, including alternative protein complexes harbouring at least one different subunit. We found that 26% of the reactions are catalysed by redundant enzymes. This level of enzyme redundancy is similar to the level reported in other bacteria like *E. coli* [10] and *Bacillus subtilis* [11], 31% and 30% respectively, or *P. aeruginosa* [12], 22 %. Additional comparisons with other species are shown in Supplementary Figure 2.

Next, we predicted the robustness $R_{(proliferation,\pi i)}$ of the biomass production, later referred as the proliferation phenotype, in front of an internal perturbation (π_i). We thereafter refer as internal perturbation any failure of internal network components leading to loss of function, like gene facing deleterious mutations, stochastic expression, or even enzymes facing loss of activity due to miss folding or inhibitions by chemicals. The simulations were carried out using the genome-scale metabolic models of *R. solanacearum*, *E. coli* [10] and *P. aeruginosa* [12]. To apply a similar genetic perturbation to each organism we determined groups of orthologs shared by the three species and present in each metabolic model using the INPARANOID algorithm [13]. Hence, the robustness of the proliferation phenotype of the three bacteria growing with D-glucose and L-glutamate as sole source of carbon was calculated. To do so, we run a BECO analysis and collected predictions for the 421 single gene knock-out perturbations corresponding to each of the 421 orthologous families identified. The $R_{(proliferation,I\pi i)}$ of *R. solanacearum* in D-glucose was found to be 0.58 whereas a value of 0.65 and 0.66 was found for *E. coli* and *P. aeruginosa*, respectively (Supplementary Figure 3). The higher robustness observed for *E. coli* and *P. aeruginosa* was tracked and founded to be mainly due to differences in the biomass equation, from 0.04 to 0.06 of robustness. For instance, the main differences between *R. solanacearum* and *E. coli* are due to the amino acid charged on their tRNA which is taken into account into the *R. solanacearum* biomass equation but not in the *E. coli*'s one.

Hence, the analysis showed that the contribution of the genetic redundancy to sustain the proliferation phenotype is almost similar in *R. solanacearum* and *E. coli* (0.10 and 0.12, respectively) whereas the contribution of pathway redundancy is only slightly lower (0.20 versus 0.25 in *E. coli*). A similar pattern was observed after simulation of growth in L-glutamate (Supplementary Figure 3).

SUPPLEMENTARY NOTE 7

Computing robustness in randomized environmental or genetic conditions

In order to compare robustness of several phenotypes in a large set of conditions without *a priori*, we generated 1000 randomized environmental conditions and 25000 randomized genetic conditions. The randomization process is presented in Supplementary Figure 16.

Each randomized condition contains a different number (called N) of activated inputs selected in a Gaussian distribution truncated by lower and upper bounds given by the modeller to assure a minimum and a maximum number of activated inputs. The selection of the N inputs is done considering a weight assigned to each input. Higher is the weight, higher is the probability to have the input in the randomized condition. Each input is duplicated according to its weight and the selection of the N distinct inputs is performed on the new set whose the elements have been mixed Then, the set of activated inputs is translated into metabolic constraints or into node states in the regulatory network depending on the nature of the input (nutrient, metabolic gene, regulator...). At last, for each randomized condition and each objective function, the optimal value of each objective function was computed and a histogram representing the distribution of the number of conditions where an objective function was possible (optimal value >0) was plotted.

This pipeline was launched four times:

- Metabolic network without regulatory network + genetic randomised conditions

- Metabolic network without regulatory network + environmental randomised conditions

- Metabolic network with regulatory network + genetic randomised conditions

- Metabolic network with regulatory network + environmental randomised conditions

The four results were compared to measure the influence of the regulatory network on the phenotypic robustness when the metabolic network is confronted to environmental or to genetic perturbations (Supplementary Figure 16).

Algorithms of randomisation and robustness computing have been respectively implemented in the Random and ROBA (ROBustness Analysis) functions in FlexFlux [5].

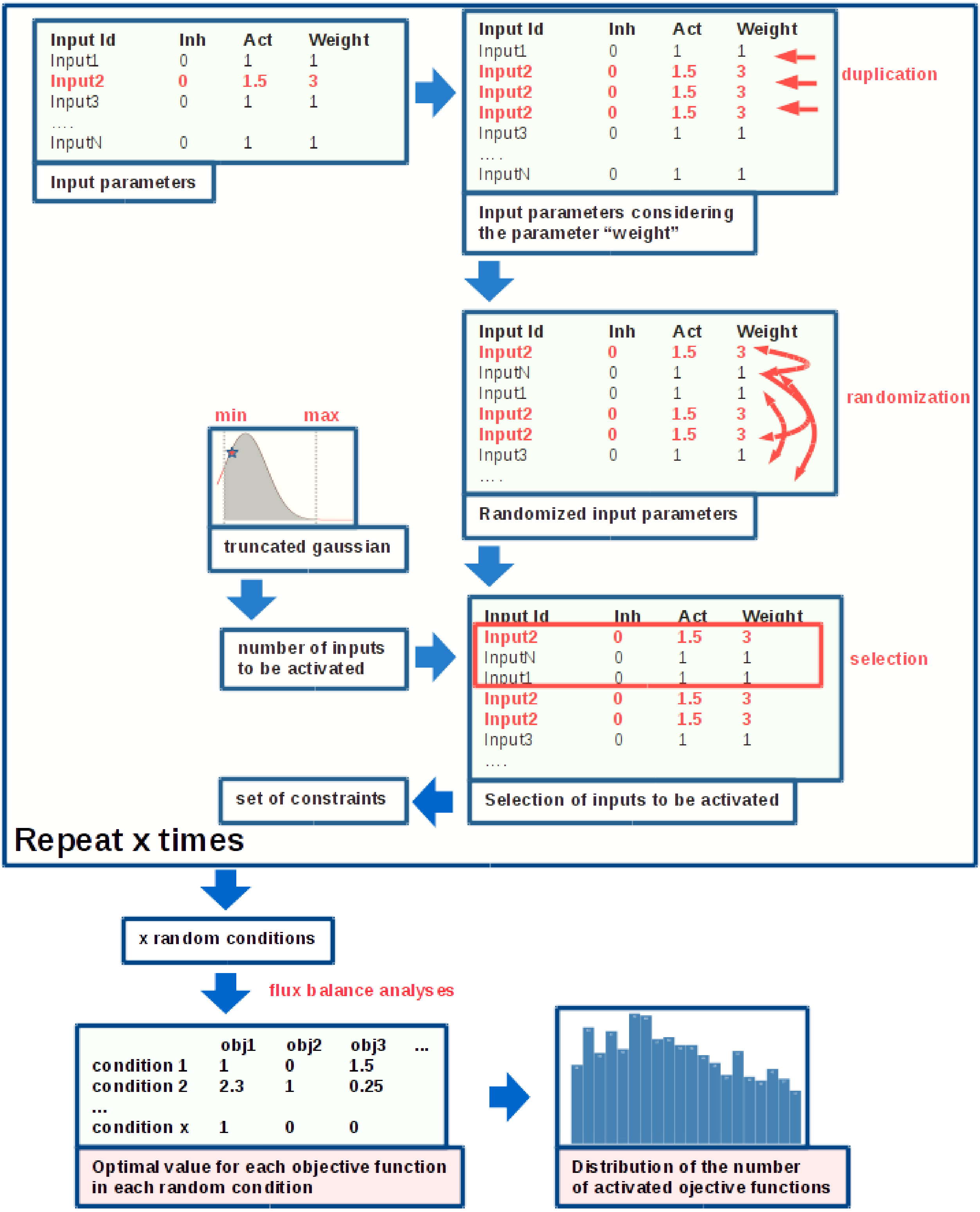

Supplementary Figure 16. Simulation to compute the robustness of cellular objectives considering randomised environments.

SUPPLEMENTARY NOTE 8

BECO analysis, gene assignment to the type of phenotypic robustness

We analyzed the 919 metabolic genes which were assigned to the different classes of phenotypic robustness by the BECO analysis. These classes correspond to pathway redundancy, genetic redundancy and versatility (Fig 5A and Supplementary Note 5). We determined how many genes in each class contributed to robustness and which of them were under control of the VRN. Results are shown for two representative phenotypic traits: one belongs to the housekeeping functions (proliferation) and the other to the virulence-associated functions (T3SS), see Fig 5B and C. These phenotypes were selected since they are dependent on a significant number of regulated genes (78) so the specific distribution of regulated genes into classes can be well discriminated from random. For the proliferation phenotype, the analysis revealed that a majority (up to 70%) of the VRN-dependent genes belongs to redundant pathways that are dependent on environmental conditions (class OPT-C and ELE-C). Such 'environment–dependent' genes can be optimal for the expression of a given phenotype in one environmental condition but not in another. On the other hand, only 10% of the VRN-controlled genes were essential genes or back-up genes for essential reactions (panels ESS and RED, respectively). Similarly to the proliferation function, the robustness of the T3SS phenotype mainly relied on redundant pathways that are dependent on environmental conditions (53 / 78 genes being controlled by the VRN). However, this analysis also showed that the VRN controlled a high number of essential genes for several virulence-associated functions and the distribution of the VRN-controlled genes in the different class for virulence-associated functions significantly differs from the distribution of gene of the proliferation function (chi-test p-value $5.2\cdot10^{-5}$). These essential genes belong to distinct virulence pathways, i.e they are different and not shared between the virulence-associated phenotypes (Supplementary Figure 17).

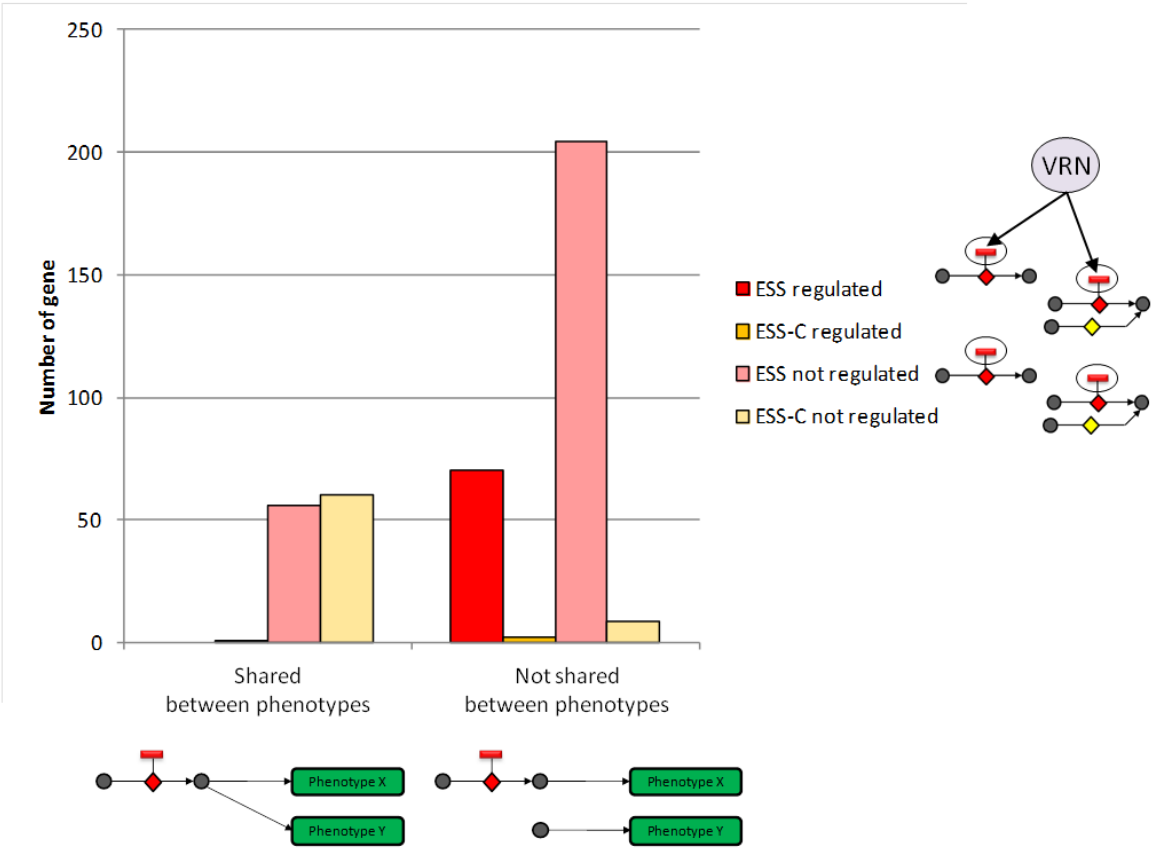

Supplementary Figure 17. Distribution of essential genes and conditionally essential genes depending of their contribution to only one phenotype (not shared between phenotypes) or multiple phenotypes (shared between phenotypes). The essential genes regulated by the VRN were found to be not shared between phenotypes.

SUPPLEMENTARY TABLE 1

	Gene in biochemical network		Gene in the VRN		Gene in genome		
Gene Product Type	Nb	% Type	Nb	% Type	Nb	% genome	Comment
o : ORF of unknown function	22	0,012	113	0,063	1784	0,313	
e : enzyme	707	0,711	85	0,085	995	0,174	
pe : putative enzyme	179	0,292	64	0,104	613	0,107	
not assigned	119	0,195	61	0,100	609	0,107	
pt : putative transporter	74	0,270	30	0,109	274	0,048	
pr : putative regulator	4	0,015	22	0,085	259	0,045	
t : transporter	156	0,653	68	0,285	239	0,042	
r : regulator	3	0,021	48	0,343	140	0,025	
f : factor	14	0,126	15	0,135	111	0,019	
pm : putative membrane component	21	0,210	20	0,200	100	0,018	
s : structure	26	0,268	32	0,330	97	0,017	
h : extrachromosomal origin	0	0,000	5	0,054	93	0,016	
ph : phenotype	71	0,866	74	0,902	82	0,014	This type includes mainly virulence associated phenotypes like TIII effectors
cp : cell process	11	0,183	13	0,217	60	0,011	
pf : putative factor	12	0,226	10	0,189	53	0,009	
lp : lipoprotein	5	0,109	11	0,239	46	0,008	
c : carrier	22	0,667	6	0,182	33	0,006	
pc : putative carrier	7	0,226	2	0,065	31	0,005	
prc : putative receptor	3	0,120	7	0,280	25	0,004	
m : membrane component	7	0,280	5	0,200	25	0,004	
ps : putative structure	10	0,417	10	0,417	24	0,004	
rc : receptor	0	0,000	9	0,750	12	0,002	
pcp : putative cell process	1	0,333	2	0,667	3	0,001	

> 30%

> 50%

Supplementary Table 1. List of fonctional categories of genes in *R. solanacearum* genome annotated in MAGE (http://www.genoscope.cns.fr/agc/microscope/home/index.php). Number and proportion of genes included in the biochemical model or the VRN are reported.

SUPPLEMENTARY TABLE 2

Species	Strain	Genus	family	Class	NC
Ralstonia solanacearum	GMI1000	Ralstonia	Burkholderiaceae	Betaproteobacteria	NC_003295.1;NC_003296.1
Ralstonia solanacearum	Po82	Ralstonia	Burkholderiaceae	Betaproteobacteria	NC_017574;NC_017575
Ralstonia solanacearum	PSI07	Ralstonia	Burkholderiaceae	Betaproteobacteria	NC_014311
Ralstonia pickettii	J12	Ralstonia	Burkholderiaceae	Betaproteobacteria	NC_010682;NC_010678;NC_010683
Ralstonia eutropha	H16	Cupriavidus	Burkholderiaceae	Betaproteobacteria	NC_008313;NC_008314;NC_005241
Cupriavidus necator	N-1	Cupriavidus	Burkholderiaceae	Betaproteobacteria	NC_015723;NC_015726;NC_015724;NC_015727
Cupriavidus metallidurans	CH34	Cupriavidus	Burkholderiaceae	Betaproteobacteria	NC_007973;NC_007974;pMOL28;pMOL30
Burkholderia cenocepacia	J2315	Burkholderia	Burkholderiaceae	Betaproteobacteria	NC_011000;NC_011001;NC_011002
Burkholderia multivorans	ATCC17616	Burkholderia	Burkholderiaceae	Betaproteobacteria	NC_010084;NC_010086;NC_010087;NC_010070
Burkholderia mallei	ATCC23344	Burkholderia	Burkholderiaceae	Betaproteobacteria	NC_006348;NC_006349
Paraburkholderia phymatum	STM815	Paraburkholderia	Burkholderiaceae	Betaproteobacteria	NC_010622;NC_010623;NC_010625;NC_010627
Polynucleobacter necessarius	QLW-P1DMWA-1	Polynucleobacter	Burkholderiaceae	Betaproteobacteria	NC_009379
Herbaspirillum seropedicae	SmR1	Herbaspirillum	Oxalobacteraceae	Betaproteobacteria	NC_014323
Massilia sp	NR 4-1	Massilia	Oxalobacteraceae	Betaproteobacteria	NZ_CP012201
Collimonas fungivorans	Ter331	Collimonas	Oxalobacteraceae	Betaproteobacteria	NC_015856
Acidovorax avenae	ATCC 19860	Acidovorax	Comamonadaceae	Betaproteobacteria	NC_015138
Comamonas testosteroni	CNB-2	Comamonas	Comamonadaceae	Betaproteobacteria	NC_013446
Bordetella pertussis	Tohama I	Bordetella	Alcaligenaceae	Betaproteobacteria	NC_002929
Achromobacter xylosoxidans	A8	Achromobacter	Alcaligenaceae	Betaproteobacteria	NC_014640
Taylorella equigenitalis	MCE9	Taylorella	Alcaligenaceae	Betaproteobacteria	NC_014914
Methylotenera versatilis	301	Methylotenera	Methylophilaceae	Betaproteobacteria	NC_014207
Thauera sp	MZ1T	Thauera	Rhodocyclaceae	Betaproteobacteria	NC_011662;NC_011667
Neisseria meningitidis	alpha-14	Neisseria	Neisseriaceae	Betaproteobacteria	NC_013016
Rhodobacter sphaeroides	ATCC17025	Rhodobacter	Rhodobacteraceae	Alphaproteobacteria	NC_009428;NC_009429;NC_009430;NC_009431;NC_009432;NC_009433
Sinorhizobium meliloti	1021	Sinorhizobium	Rhizobiaceae	Alphaproteobacteria	NC_003047;NC_003037;NC_003078
Pseudomonas syringae	DC3000	Pseudomonas	Pseudomonadaceae	Gammaproteobacteria	NC_004578
Pseudomonas aeruginosa	PAO1	Pseudomonas	Pseudomonadaceae	Gammaproteobacteria	NC_002516_2
Escherichia coli	K12	Escherichia	Enterobacteriaceae	Gammaproteobacteria	ECK
Xanthomonas campestris	ATCC33913	Xanthomonas	Xanthomonadaceae	Gammaproteobacteria	NC_003902

Supplementary Table 2. List and taxonomy of organisms used for phylogenetic analyses of the VRN-regulated genes in primary metabolism

SUPPLEMENTARY REFERENCES

1. Covert, M. W., Schilling, C. H. & Palsson, B. Regulation of gene expression in flux balance models of metabolism. J. Theor. Biol. 213, 73–88 (2001).

2. Goelzer, A. et al. Reconstruction and analysis of the genetic and metabolic regulatory networks of the central metabolism of Bacillus subtilis. BMC Syst. Biol. 2, 20 (2008).

3. Karr, J. R. et al. A whole-cell computational model predicts phenotype from genotype. Cell 150, 389–401 (2012).

4. Chaouiya, C. et al. SBML qualitative models: a model representation format and infrastructure to foster interactions between qualitative modelling formalisms and tools. BMC Syst. Biol. 7, 135 (2013).

5. Marmiesse, L., Peyraud, R. & Cottret, L. FlexFlux: combining metabolic flux and regulatory network analyses. BMC Syst. Biol. 9, (2015).

6. Jacobs, J. M., Babujee, L., Meng, F., Milling, A. & Allen, C. The in planta transcriptome of Ralstonia solanacearum: conserved physiological and virulence strategies during bacterial wilt of tomato. mBio 3, (2012).

7. Occhialini, A., Cunnac, S., Reymond, N., Genin, S. & Boucher, C. Genome-wide analysis of gene expression in Ralstonia solanacearum reveals that the hrpB gene acts as a regulatory switch controlling multiple virulence pathways. Mol. Plant-Microbe Interact. MPMI 18, 938–949 (2005).

8. Lewis, N. E. et al. Omic data from evolved E. coli are consistent with computed optimal growth from genome-scale models. Mol. Syst. Biol. 6, 390 (2010).

9. Handorf, T., Ebenhöh, O. & Heinrich, R. Expanding metabolic networks: scopes of compounds, robustness, and evolution. J. Mol. Evol. 61, 498–512 (2005).

10. Orth, J. D. et al. A comprehensive genome-scale reconstruction of Escherichia coli metabolism--2011. Mol. Syst. Biol. 7, 535 (2011).

11. Oh, Y.-K., Palsson, B. O., Park, S. M., Schilling, C. H. & Mahadevan, R. Genome-scale reconstruction of metabolic network in Bacillus subtilis based on high-throughput phenotyping and gene essentiality data. J. Biol. Chem. 282, 28791–28799 (2007).

12. Oberhardt, M. A., Puchałka, J., Martins dos Santos, V. A. P. & Papin, J. A. Reconciliation of genome-scale metabolic reconstructions for comparative systems analysis. PLoS Comput. Biol. 7, e1001116 (2011).

13. Remm, M., Storm, C. E. & Sonnhammer, E. L. Automatic clustering of orthologs and in-paralogs from pairwise species comparisons. J. Mol. Biol. 314, 1041–52 (2001).
