# Supplementary material for: Control of primary metabolism by a virulence regulatory network promotes robustness in a plant pathogen
Source: Nat Commun. 2018 Jan 29;9:418. doi: 10.1038/s41467-017-02660-4 (PMC5788922; doi:10.1038/s41467-017-02660-4)
Supplement: Supplementary file 3 — Description of Additional Supplementary Files [file 41467_2017_2660_MOESM3_ESM.pdf]

Description of Additional Supplementary Files

File Name: Supplementary Data 1
Description: **Virulence regulatory network**. Information on the VRN from *R. solanacearum* strain GMI1000.

File Name: Supplementary Data 2
Description: **Environmental conditions and compositions**. List of environmental conditions and their composition used in the study.

File Name: Supplementary Data 3
Description: **VRN validation for predicting gene expression profile**. List of experimental and simulated expression profiles and statistics.

File Name: Supplementary Data 4
Description: **List and phenotypes of deletion mutants tested**. List of mutants used for phenotypic validation and list of the associated phenotypes

File Name: Supplementary Data 5
Description: **Tn5 insertion mutant library**. Complete list of the Tn5 insertion mutants.

File Name: Supplementary Data 6
Description: **Tn5 insertion mutant phenotypes**. Tn5 mutant essentiality phenotypes on complete medium, D-glucose, L-glutamate as sole source of carbon and energy.

File Name: Supplementary Data 7
Description: **Environmental conditions used in BECO analysis**. Environmental conditions tested for the BECO analysis

File Name: Supplementary Data 8
Description: **Results of BECO analysis**. Gene number in each BECO classification categories.

File Name: Supplementary Data 9
Description: **GC content of genes involved in the hybrid model.**

File Name: Supplementary Data 10
Description: **OrthoMCL analysis of 12 VRN-regulated genes involved in primary metabolism, but not in amino acid biosynthesis pathways**.

File Name: Supplementary Software 1
Description: Virulence regulatory network model iRP1443REG. VRN model of *R. solanacearum* GMI1000, iRP1443REG, in sbml-qual format.
